# Regarding: “The effect of omega-3 fatty acid supplementation on clinical and biochemical parameters of critically ill patients with COVID-19: a randomized clinical trial”

**DOI:** 10.1186/s12967-021-02958-4

**Published:** 2021-06-30

**Authors:** Takeshi Nakata, Takashi Ariie, Shunsuke Taito

**Affiliations:** 1 Department of Endocrinology Metabolism, Rheumatology and Nephrology, Faculty of Medicine, Oita University, 1-1 Idaigaoka, Hasama-machi Yufu, Oita, 879-5593 Japan; 2 Department of Physical Therapy, School of Health Sciences at Fukuoka, International University of Health and Welfare, 137-1 Enokizu, Okawa-shi, Fukuoka, 831-8501 Japan; 3 Systematic Review Workshop Peer Support Group (SRWS-PSG), Osaka, Japan; 4 Division of Rehabilitation, Department of Clinical Practice and Support, Hiroshima University Hospital, Kasumi 1-2-3, Minami-ku, Hiroshima, 734-8551 Japan

To the Editor,

We read the article “The effect of omega-3 fatty acid supplementation on clinical and biochemical parameters of critically ill patients with COVID-19: a randomized clinical trial” with very interest [1]. We agree that omega-3 polyunsaturated fatty acid (n3-PUFA) supplementation has some clinical benefits. However, we have two methodological concerns, which may alter the conclusion.

First, this study has a high risk of bias for selection of the reporting results [2]. The authors mentioned the effectiveness of n3-PUFAs for treating metabolic acidosis and enhancing renal function, although these outcomes were not specified as the primary outcomes in the protocol [3]. In addition, the sample size of this study and that calculated in protocol were quite different. This point is important because with a small sample size, the possibility that the results were attributable to chance or bias cannot be excluded, and it is unclear whether the study was adequately powered. Therefore, the authors should provide the information regarding the primary outcome which was used for calculating the sample size and the magnitude of the difference that the study was designed to detect. These data would provide readers with a better understanding of the study.

Second, n3-PUFAs might not improve the outcome two weeks after the start of the intervention. All-cause mortality is one of the critical outcomes reported by the Core Outcome Set for Clinical Trials on Coronavirus Disease 2019 [4]. Although the authors emphasized the better results of the 1-month survival in the intervention group, the mortality at the end of 2-week treatment period was much higher in the intervention group than that of the control group (16.7% vs 9.6%). As the authors presented laboratory data collected at the end of 2 weeks of treatment, the 2-week mortality should be mentioned. Additionally, in general the results of per protocol analyses sometimes make the intervention appear to be more effective than the results of intention-to-treat analyses [5]. So, the results of a modified intention-to-treat analysis which includes data on the participants who withdrew after randomization (because of no indication for enteral feeding), the results might provide more useful knowledge for clinicians to evaluate the true effects of n3-PUFAs in an actual clinical setting.

## Data Availability

Not applicable.
